# Loss of IκB kinase β promotes myofibroblast transformation and senescence through activation of the ROS-TGFβ autocrine loop

**DOI:** 10.1007/s13238-015-0241-6

**Published:** 2016-03-05

**Authors:** Liang Chen, Zhimin Peng, Qinghang Meng, Maureen Mongan, Jingcai Wang, Maureen Sartor, Jing Chen, Liang Niu, Mario Medvedovic, Winston Kao, Ying Xia

**Affiliations:** Department of Environmental Health and Center of Environmental Genetics, University of Cincinnati Medical Center, Cincinnati, OH 45267 USA; Department of Ophthalmology, University of Cincinnati Medical Center, Cincinnati, OH 45267 USA

**Keywords:** IkB kinase β (IKKβ), nuclear factor κB (NF-κB), transforming growth factors β (TGFβ), reactive oxygen species (ROS), myofibroblast, senescence

## Abstract

Using forward and reverse genetics and global gene expression analyses, we explored the crosstalk between the I**κ**B kinase β (IKKβ) and the transforming growth factor β (TGFβ) signaling pathways. We show that *in vitro* ablation of *Ikkβ* in fibroblasts led to progressive ROS accumulation and TGFβ activation, and ultimately accelerated cell migration, fibroblast-myofibroblast transformation and senescence. Mechanistically, the basal IKKβ activity was required for anti-oxidant gene expression and redox homeostasis. Lacking this activity, IKKβ-null cells showed ROS accumulation and activation of stress-sensitive transcription factor AP-1/c-Jun. AP-1/c-Jun activation led to up-regulation of the *Tgfβ2* promoter, which in turn further potentiated intracellular ROS through the induction of NADPH oxidase (NOX). These data suggest that by blocking the autocrine amplification of a ROS-TGFβ loop IKKβ plays a crucial role in the prevention of fibroblast-myofibroblast transformation and senescence.

**Electronic supplementary material:**

The online version of this article (doi:10.1007/s13238-015-0241-6) contains supplementary material, which is available to authorized users.

## INTRODUCTION

The IκB kinase β (IKKβ) is a key catalytic subunit of the IKK complex, involved in inflammatory responses. It is robustly activated by cytokines, bacterial and viral products and metabolic stresses. IKKβ activation leads to phosphorylation of inhibitor of κB (IκB), and subsequently, translocation of the nuclear factor κB (NF-κB) to nucleus. The nuclear NF-κB binds to the κB elements in gene promoters and enhancers to either activate or repress gene expression (Perkins, 2007). By regulating genes coding for cytokines, chemokines, enzymes and molecules with microbicidal activity, the IKK-NF-κB cascade offers important protection against stress and danger signals (Vallabhapurapu and Karin, 2009). Persistent and unrestrained activation of the cascade, on the other hand, leads to chronic inflammation that may be the underlying cause of detrimental and life-threatening diseases, such as rheumatoid arthritis, atherosclerosis and cancer (Luo et al., 2005; Kim et al., 2006; Chariot, 2009). For this reason, inhibition of IKK signaling is widely considered as a promising strategy for treating many illnesses; the challenge however is to fully recognize, and develop means to offset, the potential harmful consequences of pathway inactivation (Baldwin, Jr., 2001; Li et al., 2002; Bacher and Schmitz, 2004; Courtois and Gilmore, 2006; Karin, 2006).

IKKβ maintains low static activity in the absence of external stimuli. This is associated with slow IκB degradation and equilibrium NF-κB activity (O’Dea et al., 2007). The basal activity is important for redox homeostasis, thus IKKβ inactivation renders cells or tissues vulnerable to oxidative damage (Gerondakis et al., 2006). For example, when *Ikkβ* is knocked out in hepatocytes, the livers of the knockout mice have normal development, but exhibit elevated levels of reactive oxygen species (ROS). In addition, IKKβ-defective livers are susceptible to injuries by carcinogens, concanavalin A and bacterial infection (Lavon et al., 2000; Maeda et al., 2005). When IKKβ is knocked out in fibroblasts, the null cells have elevated ROS levels and are sensitive to damage by stress and injury (Maeda et al., 2005; Chen et al., 2006; Giorgio et al., 2007; May and Madge, 2007; Sen and Roy, 2010). These observations suggest that IKKβ may be involved in a plethora of physiological processes through the regulation of redox homeostasis (Karin, 2008; Pasparakis, 2009).

In the present work, we investigated the role of IKKβ through global gene expression analyses and identified a crosstalk interaction between IKKβ and TGFβ signaling. We showed that loss of IKKβ in fibroblasts led to TGFβ activation, which in turn modulated cell motility, myofibroblast transformation and senescence. These results suggest that IKKβ can act as a repressor of the TGFβ pathway.

## RESULTS

### IKKβ represses TGFβ signaling

To explore the roles of IKK and NF-κB signaling in fibroblasts, we examined global gene expression in wild type and cells lacking IKKα, IKKβ or the p65 subunit of NF-κB. Comparison of differentially expressed genes between wild type and knockout cells, we found that genes up-regulated in the wild type cells were enriched for the terpenoid backbone biosynthesis pathway, whereas genes down-regulated in the wild type cells were enriched for the focal adhesion and vascular smooth muscle contraction pathways (Table 1).

**Table 1:** Biological pathways affected by the IKK-NF-κB cascade*

	**WT vs. Ikkα** ^***−*/*−***^	**WT vs. Ikkβ** ^−/***−***^	**WT vs. p65** ^−/***−***^
**Up-regulated genes enriched pathways**			
Terpenoid backbone biosynthesis	0.003663	1.53 × 10^−5^	0.014653
**Down-regulated genes enriched pathways**			
Focal adhesion	1.78 × 10^−8^	0.039390	3.14 × 10^−10^
Vascular smooth muscle contraction	9.00 × 10^−7^	0.000589	1.66 × 10^−5^

* Each entry is the False Discovery Rate (FDR) adjusted *P*-values for the pathway in the corresponding row in the comparison in the corresponding column. The *P*-values were calculated by R package CLEAN using the KEGG pathway database

We further examined differential gene expression between IKKβ-competent (*Ikkβ*^*−*/*−*^/Ad-IKKβ) and -deficient (*Ikkβ*^*−*/*−*^/Ad-β-Gal and *Ikkβ*^*−*/*−*^) cells using the same strategy. Genes up-regulated in the IKKβ-competent cells were, as expected, enriched for pathways involved in immunity and inflammation, such as antigen processing and presentation, rheumatoid arthritis, and B cell receptor signaling pathway and allograft rejection, but intriguingly, genes down-regulated in the IKKβ-competent cells were enriched for focal adhesion, ECM-receptor interaction and, and the TGFβ signaling pathways (Table 2).

**Table 2:** The IKKβ-regulated biological pathways*

	**Ad-IKKβ vs. uninfected**	**Ad-IKKβ vs. Ad-β-Gal**
**Up-regulated genes enriched pathways**		
Antigen processing and presentation	3.74 × 10^−6^	4.02 × 10^−6^
Leishmaniasis	3.69 × 10^−5^	3.29 × 10^−12^
Phagosome	4.00 × 10^−5^	0.002085
Rheumatoid arthritis		1.51 × 10^−9^
B cell receptor signaling pathway	0.000402	0.000641
Graft-versus-host disease	0.002208	0.002457
Allograft rejection	0.003035	0.004726
Type I diabetes mellitus	0.009985	0.022062
Autoimmune thyroid disease	0.013477	0.025376
**Down-regulated genes enriched pathways**		
Focal adhesion	0.000511	6.21 × 10^−6^
TGFβ signaling pathway	0.009496	0.028866
ECM-receptor interaction	4.73 × 10^−8^	4.70 × 10^−7^
Protein digestion and absorption	1.18 × 10^−7^	0.001664
Amoebiasis	5.40 × 10^−6^	0.000540

* Each entry is the False Discovery Rate (FDR) adjusted *P*-values for the pathway in the corresponding row in the comparison in the corresponding column. The *P*-values were calculated by R package CLEAN using the KEGG pathway database

**Figure 1:**
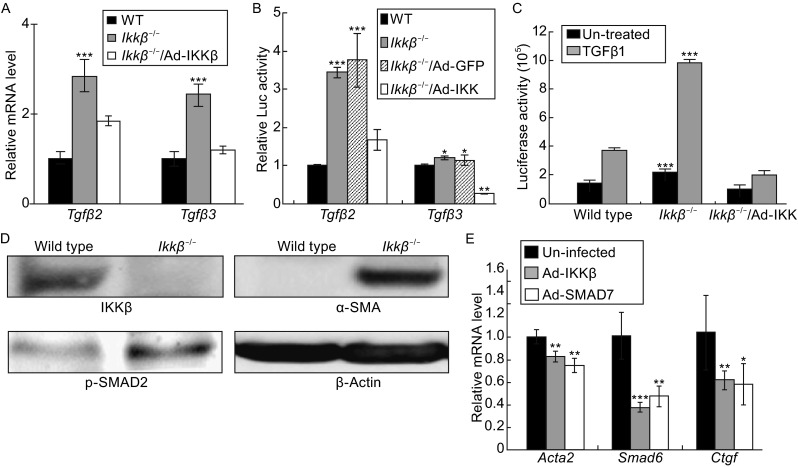
**Loss of IKKβ upregulates TGFβ expression and activity**. The IKKβ-competent, i.e. wild type and *Ikkβ* ^*−*/*−*^/Ad-IKKβ, and IKKβ-deficient, i.e. *Ikkβ* ^*−*/*−*^ and *Ikkβ* ^*−*/*−*^ */*Ad-GFP, fibroblasts were examined for *Tgfβ2* and *3* (A) mRNA expression and (B) promoter activity, and for (C) basal (un-treated) and TGFβ1-induced SMAD activity (SBE-luc) and (D) SMAD phosphorylation, and IKKβ, α-SMA and β-actin expression. (E) The *Ikkβ* ^*−*/*−*^ cells, either uninfected or infected with Ad-IKKβ and Ad-SMAD7, were examined for the expression of SMAD-target genes, i.e. *Acta2*, *Smad6* and *Ctgf*. Results represent the mean values ± SD from at least three independent experiments. *P < 0.05, **P < 0.01 and ***P < 0.001 were considered significantly different from the wild type or control samples

We validated the array data focusing on IKKβ-repressed genes of the TGFβ pathway. Compared to the wild type, the *Ikkβ*^*−*/*−*^ cells had elevated *Tgfβ2* and *Tgfβ3* mRNA transcripts (Fig. 1A), corresponding to higher gene promoter activities (Fig. 1B). They also exhibited increased SMAD transcriptional activity (Fig. 1C) and phosphorylation (Fig. 1D), as well as increased expression of a number of SMAD target genes, such as *Smad6*, *Ctgf* and *Acta2* (Figs. 1E and S1). In addition, we observed the expression of myofibroblast marker α smooth muscle actin (α-SMA), the product of *Acta2*, in IKKβ-null but not wild type cells (Fig. 1D). Adenoviral-mediated expression of IKKβ, but not of GFP used as control, in the null cells repressed *Tgfβ* expression and promoter activity, decreased SMAD activity and target gene expression, similar to the effects of Ad-SMAD7 and reached the levels same as that in the wild type cells (Fig. 1A–C and 1E). These results indicate that loss of IKKβ leads to the activation of TGFβ expression and signaling.

### TGFβ upregulation leads to migration and myofibroblast transformation of IKKβ-null cells

**Figure 2:**
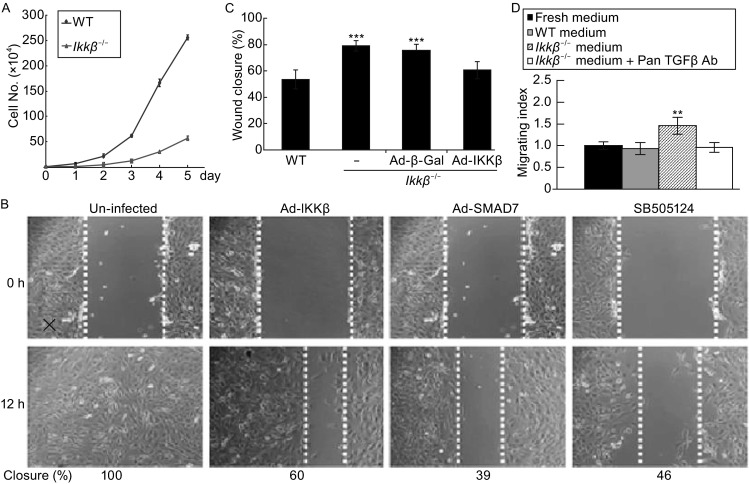
**IKKβ loss induces TGFβ expression and cell migration**. (A) The wild type and *Ikkβ* ^*−*/*−*^ fibroblasts were examined for growth rate. The *in vitro* wound healing assay was performed on wild type and *Ikkβ* ^*−*/*−*^ fibroblasts either uninfected or infected with Ad-GFP or Ad-IKKβ, or pre-treated with SB505124 for 24 h. (B) The cells were photographed at 0 and 12 h after the scratch wound, and (C) the speed of healing was calculated. (D) The *in vitro* wound healing assay was performed on wild type cells in normal growth medium, condition-medium collected from wild type cells or condition-medium collected from *Ikkβ* ^*−*/*−*^ cells with or without anti-pan-TGFβ. The healing rate was calculated at 12 h after injury. Results represent the mean values ± SD from at least three independent experiments. **P < 0.01 and ***P < 0.001 were considered significantly different from the wild type or control samples

TGFβ plays a pivotal role in cell proliferation, differentiation, wound healing and extracellular matrix production, and it induces growth arrest and myofibroblast transformation in fibroblasts (Datto et al., 1999; Phan, 2002). Chen, et al. have reported that the IKKβ-deficient cells grow slower, but migrate faster (Chen et al., 2006). We confirmed these observations (Fig. 2A and 2B), and furthermore, we showed that the migration rate of the null cells was significantly reduced by expression of IKKβ and inhibitory SMAD7, and by treatment with SB505124, a TGFβ receptor inhibitor (Fig. 2B and 2C).

To assess if promoted migration was due to TGFβ secretion, we collected conditioned medium from wild type and *Ikkβ*^*−*/*−*^ cultures and examined its effects on migration of the wild type cells. The wild type-conditioned medium had no effect, but the *Ikkβ*^*−*/*−*^-conditioned medium accelerated migration by 50% (Fig. 2D). Additionally, the migration stimulatory activity was abolished by TGFβ neutralizing antibodies, supporting the notion that TGFβ secreted by the IKKβ-null cells contributed to the stimulation of fibroblast migration.

### Progressive ROS accumulation and TGFβ activation following IKKβ ablation

**Figure 3:**
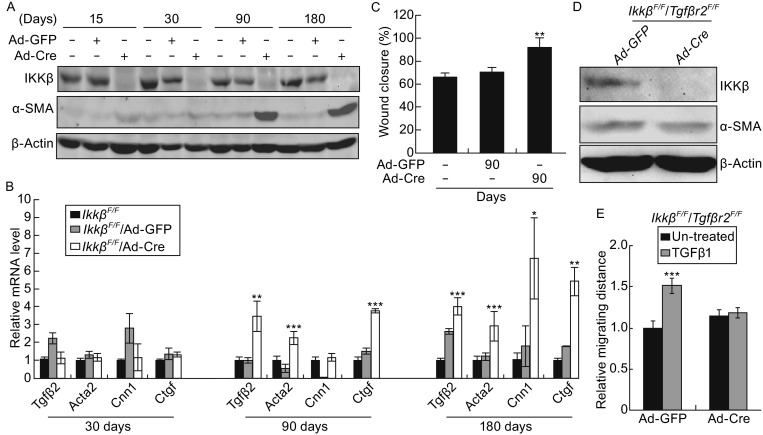
**IKKβ ablation leads to progressive activation of TGFβ and cell migration**. The *Ikkβ* ^*F*/*F*^ fibroblasts were infected with Ad-GFP or Ad-Cre and the cells were maintained in culture for various days as indicated. The cells were examined for (A) IKKβ, α-SMA and β-actin protein, (B) mRNA for *Tgfβ2* and SMAD-target genes, and (C) rate of wound healing. The *Ikkβ* ^*F*/*F*^/*Tgfbr2* ^*F*/*F*^ fibroblasts were infected with Ad-GFP or Ad-Cre. At 3 months after infection, the cells were examined for (D) IKKβ, α-SMA and β-actin protein and (E) rate of *in vitro* wound healing in the presence or absence of TGFβ1. Results represent the mean values ± SD from at least three independent experiments. *P < 0.05, **P < 0.01 and ***P < 0.001 were considered significantly different from the wild type or control samples

Infection of *Ikkβ*^*F*/*F*^ embryonic fibroblasts with Ad-Cre could ablate the *Ikkβ* gene *in vitro*. Using this approach, we generated the *Ikkβ*^*F*/*F*^/Ad-Cre cells, in which IKKβ expression, NF-κB activity, and NF-κB target gene expression were abolished or significantly reduced (Figs. 3A and S2A–S2E). Infection of the *Ikkβ*^*F*/*F*^/Ad-Cre cells with Ad-IKKβ, but not Ad-GFP, restored NF-κB activity and target gene expression (Fig. S2D and S2E).

The *Ikkβ*^*F*/*F*^/Ad-Cre cells lacked IKKβ, but surprisingly, they did not have detectable α-SMA expression immediately following Ad-Cre infection (Fig. 3A). These cells instead displayed a gradual increase in the expression of α-SMA, TGFβ2 and SMAD-target genes (Fig. 3A and 3B), and they exhibited faster migration only after 90 days of Ad-Cre infection (Fig. 3C). Simultaneous ablation of IKKβ and TGFβ receptor 2 reduced α-SMA upregulation and TGFβ1-induced migration (Figs. 3D, 3E and S3). The data derived from the *in vitro* gene ablation system suggest that IKKβ loss leads to a gradual activation of TGFβ signaling and progressive myofibroblast conversion.

### Loss of IKKβ leads to activation of the ROS-TGFβ-NOX cascade

**Figure 4:**
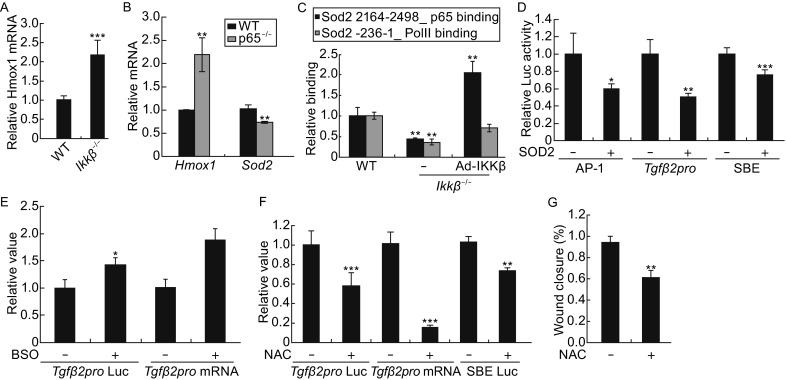
**ROS accumulation leads to TGFβ activation in IKKβ-null cells**. (A) Wild type and *Ikkβ* ^*−*/*−*^ cells and (B) wild type and *p65* ^*−*/*−*^ cells were examined for mRNA of oxidative stress marker *Hmox-1* and/or redox scavenger gene *Sod2*. (C) The wild type and *Ikkβ* ^*−*/*−*^ cells with or without Ad-IKKβ infection were examined for p65 binding of the *Sod2* enhancer and RNA Pol II occupancy of the *Sod2* promoter. (D) The *Ikkβ* ^*−*/*−*^ cells were transfected with luciferase reporters for AP-1, SMAD and *Tgfβ2* promoter, together with either an empty vector or SOD2 expression plasmids. The luciferase activities in SOD2 transfected cells were compared to those in empty vector transfected cells, designated as 1. (E) The wild type cells were either un-transfected or transfected with *Tgfβ2* promoter reporter, and treated with pro-oxidant BSO. The *Tgfβ2* mRNA expression and promoter activity were examined. The *Ikkβ* ^*−*/*−*^ cells were either un-transfected or treanfected with the luciferase reporter plasmids for *Tgfβ2* promoter or SMAD (SEB-luc). The cells were treated with anti-oxidant NAC, and were examined for (F) *Tgfβ2* mRNA expression and luciferase activity, and (G) migration by the *in vitro* wound healing assays. Results represent the mean values ± SD from at least three independent experiments. *P < 0.05, **P < 0.01 and ***P < 0.001 were considered significantly different from the wild type or control samples

Consistent with the notion that IKKβ represses reactive oxygen species (ROS) (Tanaka et al., 1999; Maeda et al., 2005), we showed that the H_2_O_2_ level, measured by 2′,7′-Dichlorodihydrofluorescein diacetate (CM-H_2_DCFDA) labeling, was high in *Ikkβ*^*−*/*−*^ but low in wild type cells (Fig. S4A). In addition, the expression of the oxidative stress-inducible biomarker gene *Heme oxygenase 1* (*Ho-1*) was more abundant in *Ikkβ*^*−*/*−*^ than wild type cells (Fig. 4A). The IKKβ- and p65-deficient cells have similar gene expression signatures and faster migration phenotype (Table 1 and Fig. S5), and like the IKKβ-null cells, the *p65*^*−*/*−*^ cells also had increased *Ho-1* expression (Fig. 4B). Furthermore, we detected in the *p65*^*−*/*−*^ cells decreased expression of superoxide dismutase 2 (*Sod2*), encoding for a crucial redox scavenger. Correspondingly, compared to the IKKβ-competent, i.e. wild type and *Ikkβ*^*−*/*−*^/Ad-IKKβ, cells, the IKKβ-deficient *Ikkβ*^*−*/*−*^cells had decreased level of RNA pol II recruitment to the *Sod2* promoter and reduced p65 bound at the gene enhancer (Fig. 4C).

To evaluate if SOD2 reduction contributed to TGFβ activation, we expressed SOD2 in IKKβ-null cells and observed a significant decrease of *Tgfβ2* promoter and SMAD activity (Fig. 4D). We further showed that SOD2 expression caused down-regulation of ROS-sensitive AP-1 activity, raising the possibility that the TGFβ signaling was actually modulated by cellular redox status (Bataller et al., 2003; Fleckenstein et al., 2007; Roy et al., 2011). To test the possibility, we treated the wild type cells with pro-oxidant L-Buthionine sulphoximine (BSO), and the *Ikkβ*^*−*/*−*^ cells with anti-oxidant N-acetyl cysteine (NAC). As predicted by the hypothesis, BSO increased *Tgfβ2* gene expression and promoter activity and NAC significantly attenuated them, and reduced SMAD activity and migration of the IKKβ-deficient cells (Fig. 4E–G). These data suggest that SOD2 reduction and ROS accumulation contribute to TGFβ activation in the IKKβ-null cells.

**Figure 5:**
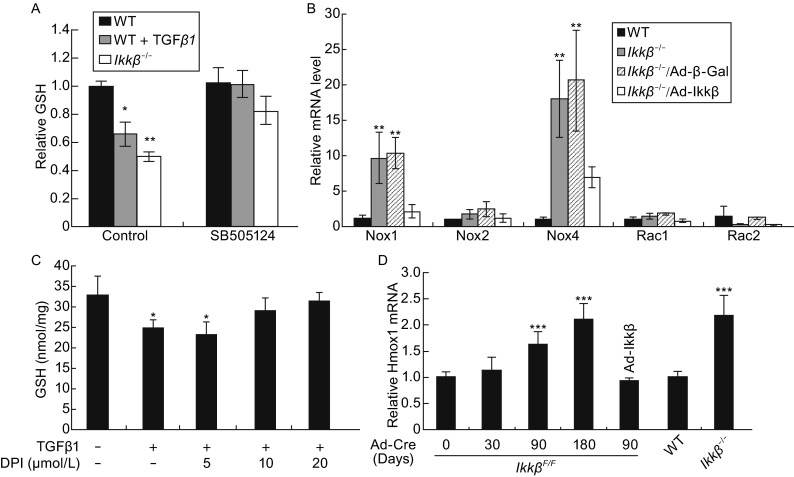
**Activation of ROS-TGFβ-NOX amplification loop in the IKKβ-null cells**. The intracellular GSH in wild type, TGFβ1 treated wild type and *Ikkβ* ^*−*/*−*^ cells, (A) in the presence or absence of the TGFβ receptor inhibitor SB505124, or (C) in the presence or absence of the NOX inhibitor DPI. (B) The mRNA of genes coding for components of the NADH complexes in IKKβ-competent, i.e. wild type and *Ikkβ* ^*−*/*−*^/Ad-IKKβ, and IKKβ-deficient, i.e. *Ikkβ* ^*−*/*−*^ and *Ikkβ* ^*−*/*−*^/Ad-β-Gal, cells. (D) The mRNA for oxidative stress marker *Hmox-1* in wild type, *Ikkβ* ^*−*/*−*^ and in *Ikkβ* ^*F*/*F*^ fibroblasts infected with Ad-Cre for 0 to 180 days with or without Ad-IKKβ. Results represent the mean values ± SD from at least three independent experiments. *P < 0.05, **P < 0.01 and ***P < 0.001 were considered significantly different from the wild type or control samples

TGFβ, on the other hand, has been shown to activate NADPH oxidases (NOX), which could further augment ROS (Hecker et al., 2009; Bondi et al., 2010). By monitoring intracellular glutathione (GSH), the most abundant redox scavenger, we observed that treating cells with TGFβ1 caused GSH depletion, whereas treating cells with TGFβ inhibitors restored GSH in IKKβ-null and TGFβ1 treated wild type cells (Anderson, 1998) (Fig. 5A). Furthermore, the IKKβ-null cells had high levels of NOX1 and NOX4 expression and NOX inhibitors abolished TGFβ-induced GSH depletion in these cells (Armstrong et al., 2002; Bedard and Krause, 2007) (Fig. 5B and 5C).

Taken together, the above data suggest a scenario that reduction of redox scavengers in IKKβ-null cells could lead to ROS accumulation; the oxidative stresses in turn might activate the TGFβ-NOX cascade to further augment ROS. IKKβ ablation therefore leads to the activation of an autocrine cycle of ROS amplification. Consistent with the conclusion, we found that Ad-Cre infection of the *Ikkβ*^*F*/*F*^ cells led to a gradual ROS increase. While 36% *Ikkβ*^*F*/*F*^/Ad-Cre cells displayed high H_2_O_2_ level at 30 days of Ad-Cre infection, the number increased to almost 50% at 90 days after infection (Fig. S4B and S4C). Similarly, the HO-1 expression increased gradually after Ad-Cre infection of *Ikkβ*^*F*/*F*^ cells, and by 180 days, it reached the levels similar to that in *Ikkβ*^*−*/*−*^ cells and twice that in wild type or Ad-IKKβ-infected *Ikkβ*^*F*/*F*^/Ad-Cre cells (Fig. 5D).

### AP-1 is involved in ROS-induced TGFβ expression

**Figure 6:**
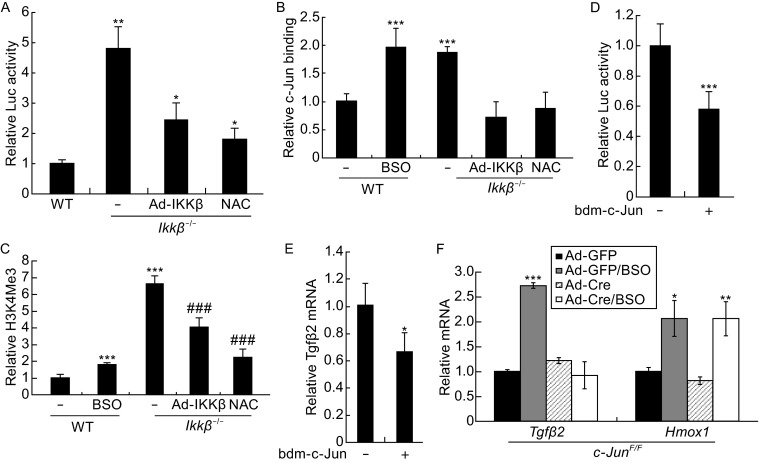
**c-Jun regulates TGFβ expression in IKKβ-null cells**. The wild type cells with or without BSO treatment, and the *Ikkβ* ^*−*/*−*^ cells with or without Ad-IKKβ infection or NAC treatment were examined for (A) luciferase activity following AP-1-luc plasmid transfection, and (B and C) ChIP assays for (B) c-Jun binding of the *Tgfβ2* enhancer and (C) H3K4me3 modification of the *Tgfβ2* promoters. The *Ikkβ* ^*−*/*−*^ cells were transfected with a dominant negative c-Jun (bdm-c-Jun) expression plasmids, and (D) together with *Tgfb2-luc* and examined for the luciferase activities, and (E) examined for the *Tgfb2* mRNA. (F) The mRNA for *Tgfb2* and *Hmox-1* was examined in *c-Jun* ^*F*/*F*^ cells infected with Ad-Cre or Ad-GFP and treated with BSO. Results represent the mean values ± SD from at least three independent experiments. *P < 0.05, **P < 0.01 and ***P < 0.001 were considered significantly different from the un-treated wild type samples; ###*P* < 0.001 was significantly different from the un-treated *Ikkβ* ^*−*/*−*^ samples

To identify the molecular link between ROS and TGFβ, we scanned the *Tgfβ2* promoter for transcription factor binding sites and found two potential AP-1-cJun binding sites (Fig. S6A). AP-1 is a stress responsive transcription factor; we tested its activation with a luciferase reporter bearing an AP-1 binding site and found that luciferase expression was induced by IKKβ ablation, but repressed by IKKβ expression and NAC treatment (Fig. 6A). In addition, AP-1 binding to the *Tgfβ2* promoter, as measured by chromatin immunoprecipitation, was increased, associated with the transcriptionally active H3K4me3 modification on the *Tgfβ2* promoter, in IKKβ-deficient cells (Fig. S6B and S6C). Both AP-1 binding and H3K4me3 were potentiated by IKKβ ablation and BSO treatment, but reduced by IKKβ over-expression and NAC treatment (Fig. 6B and 6C).

To validate the role of AP-1-c-Jun, we expressed a dominant negative mutant c-Jun (bdm-c-Jun) in the *Ikkβ*^*−*/*−*^ cells and found that its expression repressed *Tgfβ2* promoter activity and gene expression (Fig. 6D and 6E). We further used c-Jun-competent (*c-Jun*^*F*/*F*^/Ad-GFP) and -deficient (*c-Jun*^*F*/*F*^/Ad-Cre) cells and showed that while c-Jun ablation did not affect HO-1 induction, it abolished *Tgfβ2* induction under the oxidative stress conditions created by BSO treatment (Fig. 6F). Collectively, our data suggest that the ROS may act upstream to activate AP-1/c-Jun, which in turn can induce *Tgfβ* promoter and gene expression in the *Ikkβ*-null cells.

### Loss of IKKβ leads to senescence

**Figure 7:**
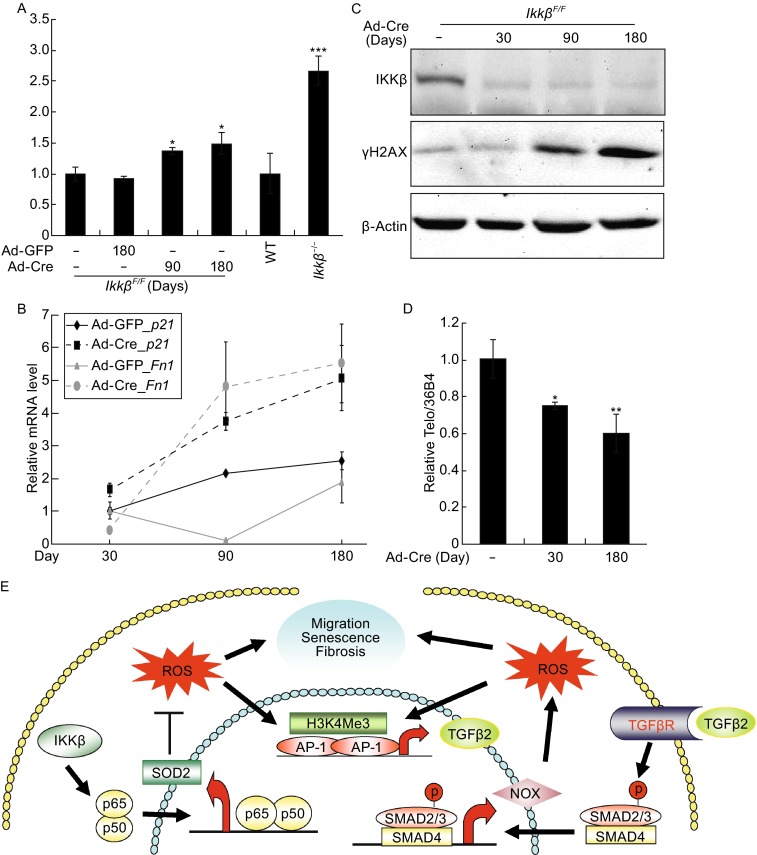
**Phenotype of the IKKβ-deficient cells**. The wild type, *Ikkβ* ^*−*/*−*,^ *Ikkβ* ^*F/F*^/Ad-GFP and *Ikkβ* ^*F*/*F*^/Ad-Cre cells were examined for (A) SA-β-gal activity, (B) expression of senescent markers, *p21* and *Fn*, (C) expression of IKKβ, β-actin and γH2AX, a marker for DNA damage, and (D) the telomere length. Results represent the mean values ± SD from at least three independent experiments. * *P* < 0.05, ** *P* < 0.01 and *** *P* < 0.001 were considered significantly different from the wild type or control samples. (E) A proposed model depicting the role of IKKβ in the regulation of the ROS-TGFβ autocrine amplification loop. Specifically, IKKβ acts through p65 to regulate expression of anti-oxidant genes, such as SOD2. Loss of IKKβ decreases SOD2 expression and dampens the scavenger capacity, resulting in ROS accumulation and AP-1/c-Jun activation. The AP-1/c-Jun regulates TGFβ expression thereby activating the TGFβ-NOX axis to further potentiate ROS accumulation. The amplification of the ROS-TGFβ-NOX axis eventually leads to increased cell migration, myofibroblast transformation and senescence

Chronic oxidative stress can induce, stabilize and amplify senescence, leading ultimately to the detrimental effects of aging (Passos et al., 2010; Nelson et al., 2012). To assess if IKKβ ablation could lead to senescence, we examined the expression of senescence-associated β-Galactosidase (SA-β-Gal) (Dimri et al., 1995). SA-β-Gal activity was low in *Ikkβ*^*F*/*F*^ cells, but gradually increased following Ad-Cre infection; by 180 days after infection the activity reached approximately 50% of the level in *Ikkβ*^*−*/*−*^cells (Fig. 7A). In Ad-Cre infected *Ikkβ*^*F*/*F*^ cells, there was also a progressive increase of the cell cycle regulator cyclin-dependent kinase inhibitor 1A (p21) (Cdkn1a), the extracellular matrix component Fibronectin (*Fn1*), and γH2AX, a histone modification associated with DNA double strand damage (Dumont et al., 2000; Debacq-Chainiaux et al., 2008; Weyemi et al., 2011) (Fig. 7B and 7C). Furthermore, there was a slight but gradual increase of telomere shortening, suggesting that IKKβ loss may lead to irreversible DNA damage and a senescent phenotype (Balaban et al., 2005; Giorgio et al., 2007) (Fig. 7D). Hence, by repressing the ROS-AP-1-TGFβ axis IKKβ may prevent senescence in fibroblasts (Fig. 7E).

## DISCUSSION

The global gene expression signatures provide an initial clue that loss of IKKβ or key components of the NF-κB pathways may lead to activation of TGFβ signaling in fibroblasts. Following this lead, we have identified a molecular link between the IKKβ and TGFβ pathways. We show that the IKKβ-NF-κB cascade sustains the expression of anti-oxidant genes and that inactivation of this cascade impedes the scavenge capacity and results in ROS accumulation. Elevated ROS in turn triggers the feed-forward activation of the ROS-AP-1-TGFβ-NOX loop that leads ultimately to increased motility, fibroblast-myofibroblast transformation, and senescence (Fig. 7E).

The antagonistic relationship between IKK and TGFβ signaling has been reported in other experimental settings. For example, in osteoclasts and head and neck cancers, TGFβ is found acting through the TGFβ activated kinase 1 to activate IKK and NF-κB, whereas NF-κB up-regulates SMAD7 to inhibit TGFβ signaling (Gingery et al., 2008; Freudlsperger et al., 2013). The IKK-NF-κB pathway is also found to modulate transcription factors/cofactors and attenuate SMAD activity (Dennler et al., 2000; Nagarajan et al., 2000; Bitzer et al., 2000; Verrecchia et al., 2001). Here we describe a unique mechanism where the crosstalk of IKK and TGFβ is mediated by ROS. Specifically, the elevated ROS in IKKβ-null cells induce c-Jun binding and activation of the *Tgfβ* promoter.

There are at least two sources for the ROS in IKKβ-null cells. First, IKKβ ablation results in insufficient ROS removal due to down-regulation of antioxidant genes, in agreement with previous reports (Chen et al., 2003; Sakon et al., 2003; Peng et al., 2007; Peng et al., 2010). Second, IKKβ ablation causes increased ROS production as the result of TGFβ induced NOX4 expression and NADH activity. Interestingly, the TGFβ-NOX axis itself is also activated by ROS, and thus, this axis and ROS may form an autocrine loop to amplify each other. Such feed-forward signal amplification is likely to be responsible for the progressive ROS accumulation and TGFβ activation in fibroblasts following IKKβ ablation. When the TGFβ signals reach a threshold level, it is able to induce cell migration and myofibroblast transformation; when the chronic ROS reach a threshold level, they may contribute to premature senescence, as it also happens in cells deficient in GSH (Chen et al., 2009).

As the IKK-NF-κB cascade is a major player of the inflammatory response, its inhibition is a promising strategy for treating a vast number of diseases associated with inflammation (McIntyre et al., 2003; Ruocco et al., 2005; Polzer et al., 2008). In particular, this cascade is considered a molecular link between inflammation and cancer; therefore, targeting the cascade has become an attractive rationale in cancer therapy (Vallabhapurapu and Karin, 2009; DiDonato et al., 2012). The caveat is that such treatment may have adverse effects due to disruption of the cascade’s pleiotropic physiological functions (DiDonato et al., 2012). Our data in fibroblasts echo this concern and suggest that complete, irreversible and long-term inhibition of IKKβ may lead to chronic oxidative stress, and increase the risks for fibrogenesis and senescence.

## MATERIALS AND METHODS

### Viruses, plasmids, reagents and antibodies

The adenoviral expression vectors for IKKβ, SMAD7, β-GAL, GFP and GFP-Cre were from Drs. Yi Zheng at the Cincinnati Children’s Hospital, Yinling Hu at the National Cancer Institute, and Chia-yang Liu at Indiana University. The reporter plasmids, NF-κB-luc, SBE-Luc, AP-1-Luc, and the *Tgfβ1*, *Tgfβ2* and *Tgfβ3* promoter-luc were obtained from Drs. Edward B. Leof at Mayo Clinic and Alvaro Puga at the University of Cincinnati (Tojima et al., 2000). Expression vector for SOD2 was from Dr. Shanglin Shi at the University of Kentucky and Bdm-c-Jun was described before (Geh et al., 2011). TGFβ1 was from PeproTech, NAC, BSO and DPI were from Sigma-Aldrich, and SB505124 was from EMD Millipore. The following antibodies were used in the study: anti- IKKα, -IKKβ, -IkBα, and -p-SMAD2 (Ser-465, 467) from Cell Signaling, anti-pan TGFβ from R&D Systems, anti-α-SMA from Abcam, anti-β-actin from Sigma-Aldrich, anti-γH2AX from Novus Biologicals, anti-PolII, -H3, -H3K27Me3, H3K9Me2, H3K9Ac and H3K4Me3 from EMD Millipore, and anti-p65, -c-Jun, and IgG from Santa Cruz Biotechnologies.

### Mouse fibroblasts, cell culture, transfection, infection and luciferase assays

The wild type, fibroblasts deficient in IKKβ, IKKα and p65 were gifts from Drs. Karin and Zandi, and were maintained under culture conditions as described (Chen et al., 2006). The *Ikkβ*^*F*/*F*^, *Ikkβ*^*F*/*F*^/*Tgfbr2*^*F*/*F*^ and *c-Jun*^*F*/*F*^ fibroblasts were prepared using E13.5 embryos following standard 3T3 protocol (Aaronson and Todaro, 1968). The cells were cultured in DMEM supplemented with 10% FBS, 50 U/mL penicillin, 50 mg/mL streptomycin for less than 10 passages before used for experiments or adenoviral infection. Some of adenoviral infected cells were allowed to grow for 6 months with approximately 50 passages. Adenoviruses were used at 100–500 PFU to infect 70% confluent cells as described before (Peng et al., 2010). Cells were transfected using the lipofectamin plus method and Firefly and Renilla luciferase activities were measured 24 to 48 h after transfection following the manufacture’s protocols (Thermo Fisher Scientific).

### Western blotting, ROS measurements, SA-β-Gal activity and *in vitro* wound healing assays

The SA-β-Gal activities were measured at the PH 6.0 using Beta-Glo Assay system (Promega), Western blotting, measurement of ROS and GSH, and the *in vitro* wound healing assays were done as previously described (Zhang et al., 2003; Peng et al., 2010). The conditional medium used for *in vitro* wound healing assays was derived from fresh medium overlaid on wild type or *Ikkβ*^*−/−*^ cells for 24 h.

### RNA isolation, reverse transcription and gene expression profiling

RNA was extracted, labeled and hybridized to Affymetrix Mouse Genome 430 2.0 Arrays using standard protocol (Medvedovic et al., 2009). Data was processed by performing background correction, quantile normalization, and calculation of expression set summaries using the Robust Multichip Average (RMA) protocol (Irizarry et al., 2003) as implemented in the Bioconductor affy package. Differentially expressed genes between two groups were identified by two-group comparison using intensity-based empirical Bayes method (IBMT) (Sartor et al., 2006). Pathway enrichment analysis was performed using the LRpath methodology (Sartor et al., 2009) implemented in the CLEAN package (Freudenberg et al., 2009).

### Quantitative RT-PCR (qRT-PCR), chromatin-immunopreciptation (ChIP) and telomere measurement

qRT-PCR was performed using a DNA Engine Opticon2 Real-Time PCR Detection System (MJ Research) and SYBR Green qPCR MasterMix (Applied Biosystems) and primers for the genes of interest as listed in Table S1. All experiments were performed at least in triplicates. The relative differences in qRT-PCR among samples were determined by the ΔCT value as described previously (Schnekenburger et al., 2007). Hence, the ΔCT value for each sample was calculated by subtracting cycle threshold (CT) value (obtained from the means of replicates) of the input DNA (or *Gapdh* signal) from that of each sample in order to normalize ChIP assay (or to normalize gene expression) results. The ΔΔCT value was calculated by subtracting control ΔCT values from the corresponding experimental ΔCT values. The resulting values were converted to fold changes over control by raising 2 to the power of −ΔCT values.

ChIP was performed following the protocol described previously (Schnekenburger et al., 2007). Briefly, cells were fixed for 10 min with 1% formaldehyde, followed by addition of 0.125 mol/L glycine for 5 min to stop cross-linking. Cells were washed with ice-cold PBS and harvested in cell lysis buffer (5 mmol/L PIPES [pH 8.0], 85 mmol/L KCl, 0.5% NP-40, and protease inhibitor cocktail [Roche]) for 10 min on ice. The nuclei were pelleted, resuspended in nucleus lysis buffer (50 mmol/L Tris-HCl [pH 8.1], 10 mmol/L EDTA, 1% SDS, and protease inhibitor cocktail), and incubated on ice for 10 min. Chromatin was sheared to a size range of 0.3 to 0.8 kb by sonication. After centrifugation to remove cell debris, chromatin was precleared for 1 h at 4°C with a 50% gel slurry of protein A-agarose beads saturated with salmon sperm DNA (Upstate), and then diluted three times in IP dilution buffer (16.7 mmol/L Tris-Cl [pH 8.1], 167 mmol/L NaCl, 1.2 mmol/L EDTA, 1.1% Triton X-100, 0.01% sodium dodecyl sulfate) with 10% of the supernatants used as input. The diluted chromatin was incubated with antibodies specific for the proteins of interest for 2 h at 4°C, followed by addition of a 50% gel slurry of protein A-agarose and incubation overnight (Upstate). The agarose beads were pelleted and washed twice with 1× dialysis buffer (50 mmol/L Tris-HCl [pH 8.0], 2 mmol/L EDTA, 0.2% Sarkosyl) and four times with IP wash buffer (100 mmol/L Tris-HCl [pH 9.0], 500 mmol/L LiCl, 1% NP-40, 1% deoxycholic acid). Precipitated chromatin complexes were removed from the beads by incubation with elution buffer (50 mmol/L NaHCO3, 1% SDS) with mild vortexing. This step was repeated, and the eluates were combined. Cross-linking was reversed by adding NaCl to a final concentration of 0.3 mol/L and incubating overnight at 65°C in the presence of RNase A. Samples were then digested with proteinase K at 45°C for 1.5 h. DNA was purified by chromatography on QIAquick columns (QIAGEN) and eluted in double-distilled water for further qPCR analysis.

The telomere length was measured using Q-PCR as described (Callicott and Womack, 2006). Briefly, the genomic DNA were extracted using a QIAmp DNA micro Kit (Qiagen, Valencia, CA, USA) and quantified.

PCR reactions were performed on the ABI Prism 7700 Sequence Detection System (Applied Biosystems), using telomeric primers for the reference control gene (mouse 36B4 single copy gene). The telomere signal was normalized to the signal from the single-copy gene to generate a relative telomere to single copy gene (T/S) ratio indicative of relative telomere length. Equal amounts of DNA (300 pg) were used for each reaction with several repeats and average telomere length was calculated.

## Electronic supplementary material

Below is the link to the electronic supplementary material.

Supplementary material 1 (PDF 772 kb)
