# Supplementary material for: Loss of IκB kinase β promotes myofibroblast transformation and senescence through activation of the ROS-TGFβ autocrine loop
Source: Protein Cell. 2016 Mar 5;7(5):338–50. doi: 10.1007/s13238-015-0241-6 (PMC4853320; doi:10.1007/s13238-015-0241-6)
Supplement: Supplementary file 1 — Supplementary material 1 (PDF 772 kb) [file 13238_2015_241_MOESM1_ESM.pdf]

Table S1. Primer sequences

Primers for quantitative PCR	
Tgfβ1 Forward	5'-AACCGCACTGTCATTCACC -3'
Tgfβ1 Reverse	5'-CGCCAA ACTTCTCCA AACC -3'
*Tgfβ2*Forward	5'-CTCAACACACCAAAGTCCTC -3'
*Tgfβ2*Reverse	5'-ATCAAAACTCCCTCCCTCC -3'
Tgfβ3 Forward	5'-ACCAAAGCGACAGACCTCAC -3'
Tgfβ3 Reverse	5'-TTCCCCTAACCAACCCACAC -3'
Tgfβr1 Forward	5'-AACCGCACTGTCATTCACC -3'
Tgfβr1 Reverse	5'-CGCCAA ACTTCTCCA AACC -3'
Tgfβr2 Forward	5'-AAGGAA AAGAAAAGGGCGG -3'
Tgfβr2 Reverse	5'-GCACACGGTAGCAGTAGA AG -3'
Thbs1 Forward	5'-TGCCCAAAGGGAGAAAGTCCAGAA -3'
Thbs1 Reverse	5'-CTGAGCAGCACACACAGAAGCATT -3'
Acta2 Forward	5'-TTGCTGACAGGATGCAGAAGGAGA -3'
Acta2 Reverse	5'-TCACAGTTGTGTGCTAGAGGCAGA -3'
Cnn1 Forward	5'-TTGTGGATGTGACAGCAGCGTTTG -3'
Cnn1 Reverse	5'-TGTATCACATGGCCTGTGTGTGGA -3'
D0H4S114 Forward	5'-AGGCCTTGTTCTGAACTGTTTGCG -3'
D0H4S114 Reverse	5'-GCAGCGCACACTGCAATTCTACAT -3'
Smad6 Forward	5'-ACCGGGTTACTCCATCAAGGTGTT -3'
Smad6 Reverse	5'-GCCGCATTGCTATCTGTGGTTGTT -3'
Ctgf Forward	5'-GACAGCTTGTGGCAAGTGAA -3'
Ctgf Reverse	5'-TTCCTCGTGGAAATCTGACC -3'
Hmox1 Forward	5'-AGGTGATGCTGACAGAGGAACACA -3'
Hmox1 Reverse	5'-ACAGAGAGAAGGCCACATTGGACA -3'
p21 Forward	5-'TTGTCGCTGTCTTGCACTC-3'
p21 Reverse	5-'AGGTTTGGAGACTGGGAGAG-3'
Fn1 Forward	5'-TTTTGACAACGGGAAGCATTATCAGATAA -3'
Fn1 Reverse	5'-TGATCAAAACATTTCTCAGCTATTGG -3'
Ccl2 Forward	5'-TCACCTGCTGCTACTCATTCACCA -3'
Ccl2 Reverse	5'-AAAGGTGCTGAAGACCTTAGGGCA -3'
Ccl7 Forward	5'-CCCAATGCATCCACATGCTGCTAT -3'
Ccl7 Reverse	5'-TGCTTCTTGGCTCCTAGGTTGGTT -3'
Il-6 Forward	5'-ACCACTTCACAAGTCGGAGGCTTA -3'
Il-6 Reverse	5'-TGGTACTCCAGAAGACCAGAGGAA -3'
IκBα Forward	5'-ATCCTGACCTGGTTTCGCTCTTGT -3'
IκBα Reverse	5'-ACACAGTCATCATAGGGCAGCTCA -3'
RelB Forward	5'-GCGGATTTGCCGAATCAACAAGGA -3'
RelB Reverse	5'-AACACATTGACAGTCACGGGCTCT -3'
Mt1 Forward	5'-AGCTTCACCAGATCTCGGAATGGA -3'
Mt1 Reverse	5'-ACGCTGGGTTGGTCCGATACTATT -3'
Mt2 Forward	5'-TGGCCATATCCCTTGAGCCAGAAA -3'
Mt2 Reverse	5'-TTGTCGGAAGCCTCTTTGCAGATG -3'
Sod1 Forward	5'-TGGCGATGAAAGCGGTGTGC -3'

***Sod1* Reverse**	**5'-GCGGCTCCCAGCATTTCCAG -3'**
***Sod2* Forward**	**5'-GCACATTAACGCGCAGATCA -3'**
***Sod2* Reverse**	**5'-AGCCTCCAGCAACTCTCCTT -3'**
***Gapdh* Forward**	**5'-AACGACCCCTTCATTGACC -3'**
***Gapdh* Reverse**	**5'-TGAAGACACCAGTAGACTCC -3'**
***Nox1* Forward**	**5'-TGGATGGATCTCTCGCTTCT-3'**
***Nox1* Reverse**	**5'-TCACCCAAGCTCTCCTCTGT-3'**
***Nox2* Forward**	**5'-CTTTCTCAGGGGTTCCAGTG-3'**
***Nox2* Reverse**	**5'-TGCAGTCTATCATCCAAGC-3'**
***Nox4* Forward**	**5'-CCAGAATGAGGATCCCAGAA-3'**
***Nox4* Reverse**	**5'-CAGCAGCAGCATGTAGAAGAC-3'**
***Rac1* Forward**	**5'-TATGGGACACAGCTGGACAA-3'**
***Rac1* Reverse**	**5'-TTGAGTCCTCGCTGTGTGAC-3'**
***Rac2* Forward**	**5'-CATCAGCTACACCACCAACG-3'**
***Rac2* Reverse**	**5'-TCTCGATGGTGTCCTTGTCA-3'**
**Primers used in CHIP**	
***Tgfβ2* promoter -1143 - -813 Forward**	**5'- CTGCAACAACAGACCTCACTTCCT -3'**
***Tgfβ2* promoter -1143 - -813 Reverse**	**5'- TCTTTCTTCCCACTGGTCTTTCCC -3'**
***Tgfβ2* promoter -870 - -370 Forward**	**5'- TCGTGGCCACAGAGTCATCACTAA -3'**
***Tgfβ2* promoter -870 - -370 Reverse**	**5'- ACACACACACACACACACACACAC -3'**
***Tgfβ2* promoter -189 - 94 Forward**	**5'- ACTGCAAATTCCTCATGCCAGTCG -3'**
***Tgfβ2* promoter -189 – 94 Reverse**	**5'- TCTCAGCGCCTTCTCTCTTTCTCT -3'**
***Mt1* promoter -183 - 168 Forward**	**5'- TGGGTTTACGGAGATAGCTGGCTT -3'**
***Mt1* promoter -183 - 168 Reverse**	**5'- ACGTTGAAGTCGTGGTGACGCTTA -3'**
***Sod2* promoter -236 - 1 Forward**	**5'- AATTCTGACCAGCAGCAGAGCCTT -3'**
***Sod2* promoter -236 - 1 Reverse**	**5'- TGTGCCAAATTGGTAGAGGCCG -3'**
***Sod2* intron 2161 – 2498 Forward**	**5'- TGTGGTTGCTGGACCTTTGGAAGA -3'**
***Sod2* intron 2161 - 2498 Reverse**	**5'- AGGAAATGCTTTCCCAACTGGGCT -3'**
**Primers used for bisulfite sequencing**	
***Tgfβ2* BS Forward**	**5'- AAGAGTTATTTTGGAGTAATTTATTTG -3'**
***Tgfβ2* BS Forward**	**5'- ACTACCTACAAAAAAAACCAACCTC -3'**
**Primers used for measurements of mouse telomere**	
Mouse telomere F	**5'-CGGTTTGTTTGGGTTTGGGTTTGGGTTTGGGTTTGGGTT-3'**
Mouse telomere R	**5'-GGCTTGCCTTACCCTTACCCTTACCCTTACCCTTACCCT-3'**
Mouse 36B4 F	**5'-ACTGGTCTAGGACCCGAGAAG-3'**
Mouse 36B4 R	**5'-TCAATGGTGCCTCTGGAGATT-3'**

Figure S1. Up-regulation of TGFβ-target genes in the IKKβ-null cells. RNA isolated from wild type and $Ikk\beta^{-/-}$ cells were examined by real-time qRT-PCR for expression of genes of the TGFβ pathway. Fold change over uninfected cells was calculated based on data from biological triplicates. Results represent the mean values ± SD from at least two independent experiments. **p<0.01 and *** p<0.001 was considered significant.

Figure S2. Induction of IKKβ loss *in vitro*. The $Ikk\beta^{F/F}$ fibroblasts were infected with Ad-GFP and Ad-Cre with or without co-infection with Ad-IKKβ for 48 h. (A) The infected cells were photographed under light and fluorescence microscopy. More than 90% $Ikk\beta^{F/F}$/Ad-GFP cells were shown to be GFP positive. (B) The $Ikk\beta^{F/F}$, $Ikk\beta^{F/F}$/Ad-Cre (infected for 48 h) and $Ikk\beta^{-/-}$ cells were treated with TNFα for various times; cell lysates were analyzed by Western blotting for IKKβ, IκBα and β-actin. The un-infected or infected $Ikk\beta^{F/F}$ cells were used for (C) RNA isolation and analyzed by real-time RT-PCR for *Ikkβ* expression, and (D) transfection with κB-Luc reporter and β-gal expression plasmid. The Luc and β-gal activities were examined 24 h after transfection, and the relative Luc activity (versus β-gal activity) was calculated. Fold change over uninfected cells was calculated based on data from biological triplicates. (E) After Ad-Cre infection, the $Ikk\beta^{F/F}$/Ad-Cre cells were passage *in vitro* for various lengths of time and without or with IKKβ re-expression (Ad-IKKβ infection). RNA was isolated and analyzed by real-time RT-PCR for the expression of NF-κB-target genes. Results represent the mean values ± SD from at least two independent experiments. *** p<0.001 was considered significant.

Figure S3. Genotyping of $Ikk\beta^{F/F}/Tgf\beta r2^{F/F}$/Ad-GFP and $Ikk\beta^{F/F}/Tgf\beta r2^{F/F}$/Ad-Cre fibroblasts. Genomic DNAs were used for PCR genotyping, using primers recognizing the $Ikk\beta^{F}$, $Ikk\beta^{\Delta}$, $Tgf\beta r2^{F}$ and $Tgf\beta r2^{\Delta}$ alleles, and primers for *Gapdh*. The PCR products were examined on agarose gel after electrophoresis.

Figure S4. Induction of IKKβ loss leads to progressive ROS elevation. (A) The wild type cells with and without H_2O_2 (100 uM) treatment for 6 h and the *Ikkβ(-/-)* cells with or without Ad-IKKβ infection for 24 h, and (B) the $Ikk\beta^{F/F}$ cells with or without Ad-Cre infection, were labeled with CM-H_2DCFDA and analyzed by flow cytometry at 485/530 nm. y axis, cell numbers, x axis, fluorescence intensity. (C) The cells were divided to two groups based on the level of DCF intensity: the DCF-high (M2) and DCF-low (M1). The percentage of cells in each group was calculated. Results represent the mean values ± SD from at least two independent experiments. *: $p<0.05$ was considered significant.

Figure S5. Increased TGFβ target gene expression and migration of the p65-null cells. (A) RNA isolated from wild type and $p65^{-/-}$ fibroblasts were analyzed by RT-PCR for selected genes of the TGFβ pathways. (B) The cells were subjected to *in vitro* wound healing assays, and healing rate was calculated by comparison of the wound sizes at 0 and 12 h of wounding. Results represent the mean values ± SD from at least two independent experiments. ***: $p <0.001$ were considered significant.

Figure S6. IKKβ loss induces c-Jun binding and H3K4Me3 modification of the *Tgfβ2* promoter (A) Illustration of approximate locations of potential AP-1 binding sites in the *Tgfβ2* promoter. The $Ikk\beta^{F/F}$ and $Ikk\beta^{F/F}$/Ad-Cre cells were subjected to Chromatin-immunoprecipitation (ChIP) assay using antibodies for (B) transcription factors, p65, p50, Nrf2 and c-Jun, and (C) histone markers, H3K27Me3, H3K9Me2 and H3K4Me3. The precipitates were examined by PCR using primers for various regions of the *Tgfβ2* promoter. The data were calculated as percentage of input and represented the mean values ± SD from at least two independent experiments. *** $p <0.001$ were considered significant.

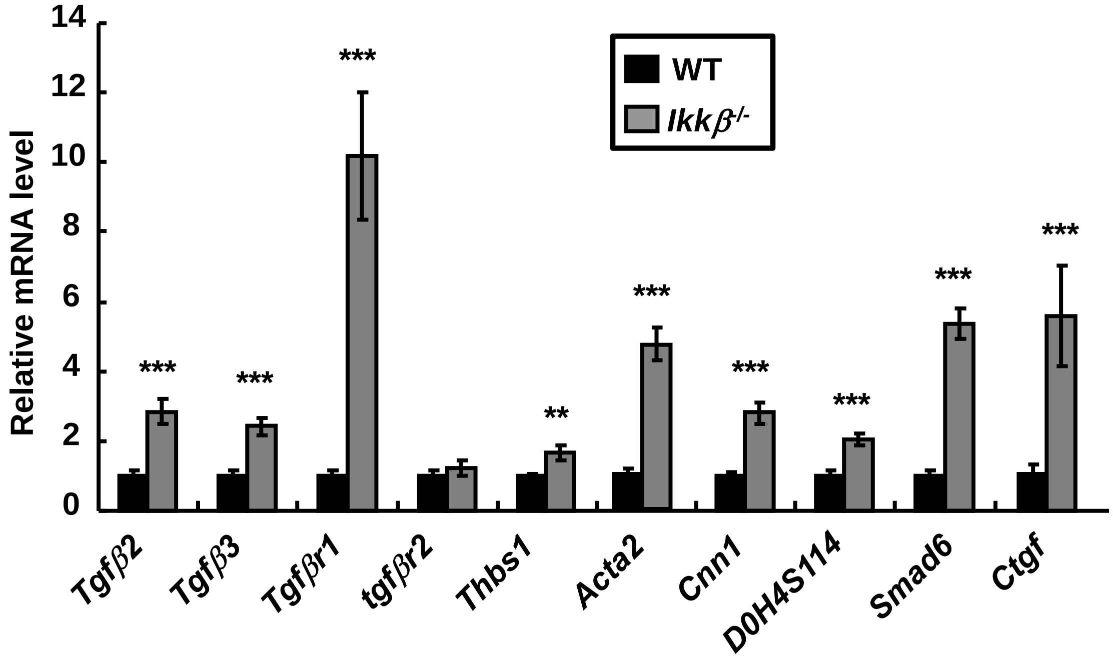

Chen, et. al. Supplemental Figure 1

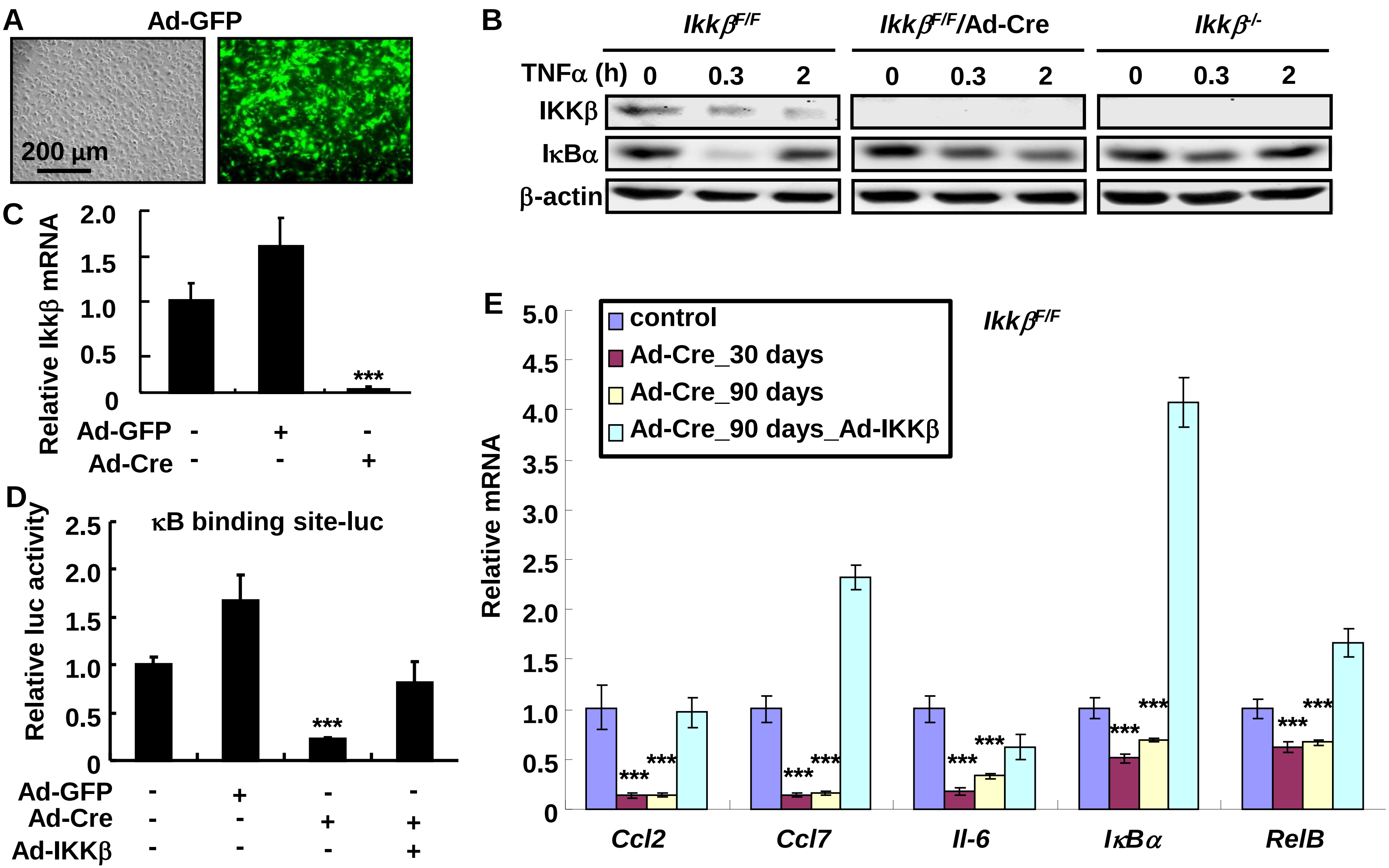

Chen, et. al. Supplemental Figure 2

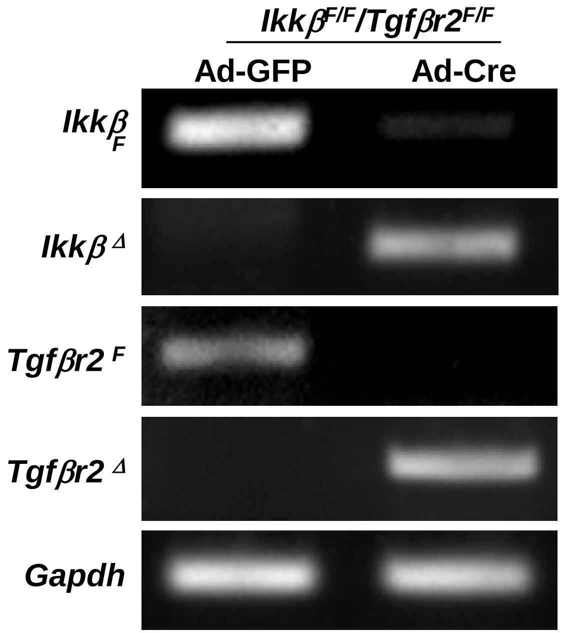

Chen, et. al. Supplemental Figure 3

A

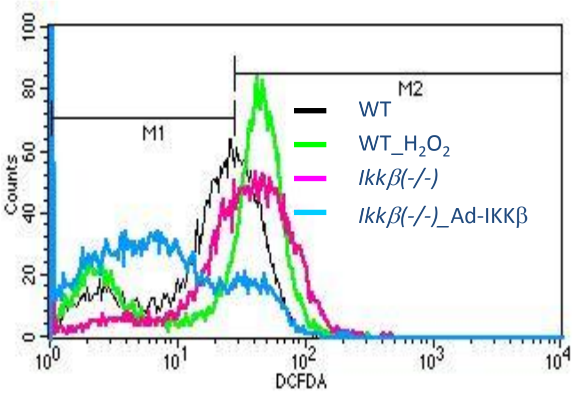

B

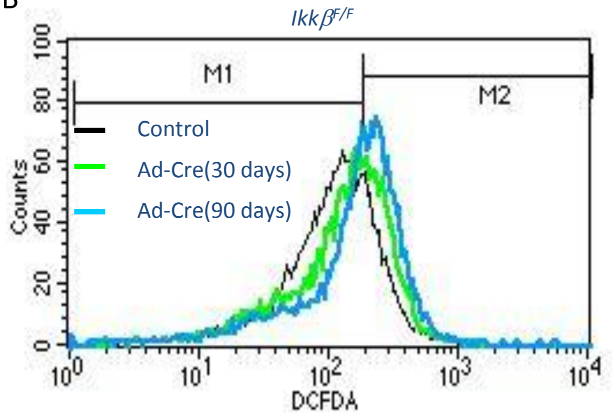

C

	M1 (Low DCFDA)	M2 (High DCFDA)
**WT**	**73.35%**	**24.12%**
**WT_H_2O_2**	**44.06%**	**53.32%**
Ikkβ(-/-)	**47.50%**	**50.54%**
***Ikkβ(-/-)*_Ad-IKKβ**	**87.90%**	**11.33%**
Ikkβ$^{F/F}$	**73.41%**	**24.02%**
***Ikkβ$^{F/F}$*_Ad-Cre_30 days**	**61.14%**	**36.07%**
***Ikkβ$^{F/F}$*_Ad-Cre_90 days**	**47.07%**	**49.57%**

Chen, et. al., Supplemental Figure 4

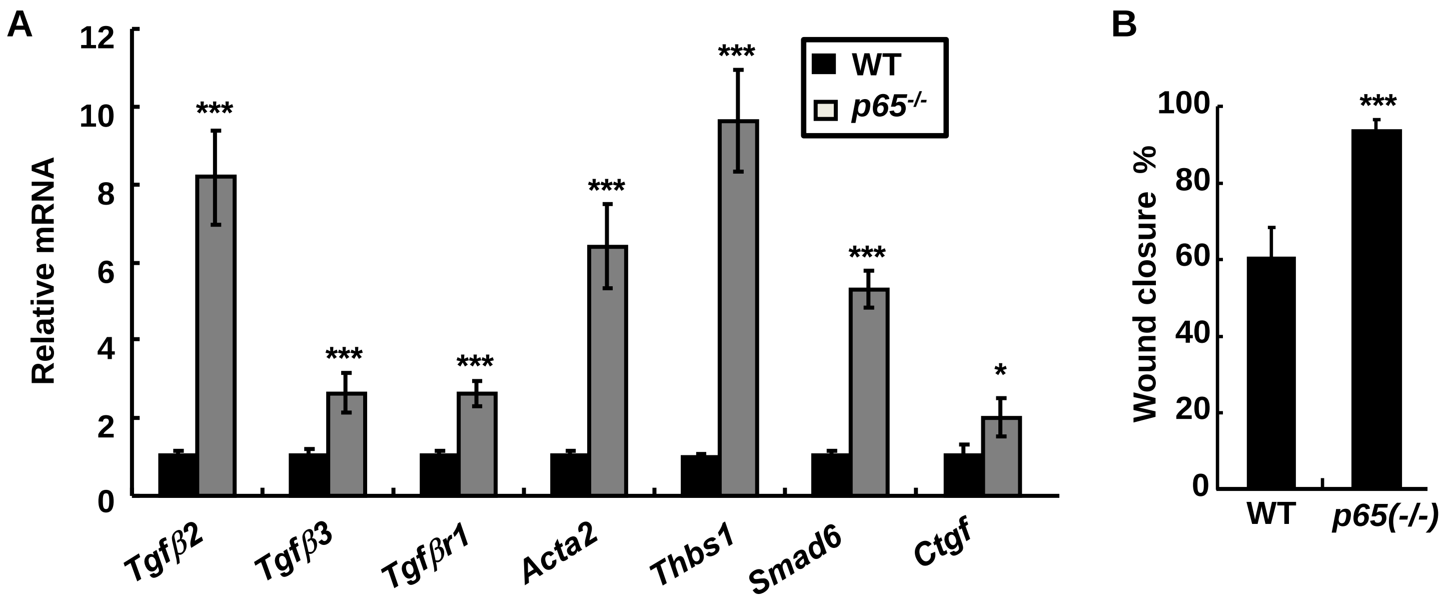

Chen, et. al., Supplemental Figure 5

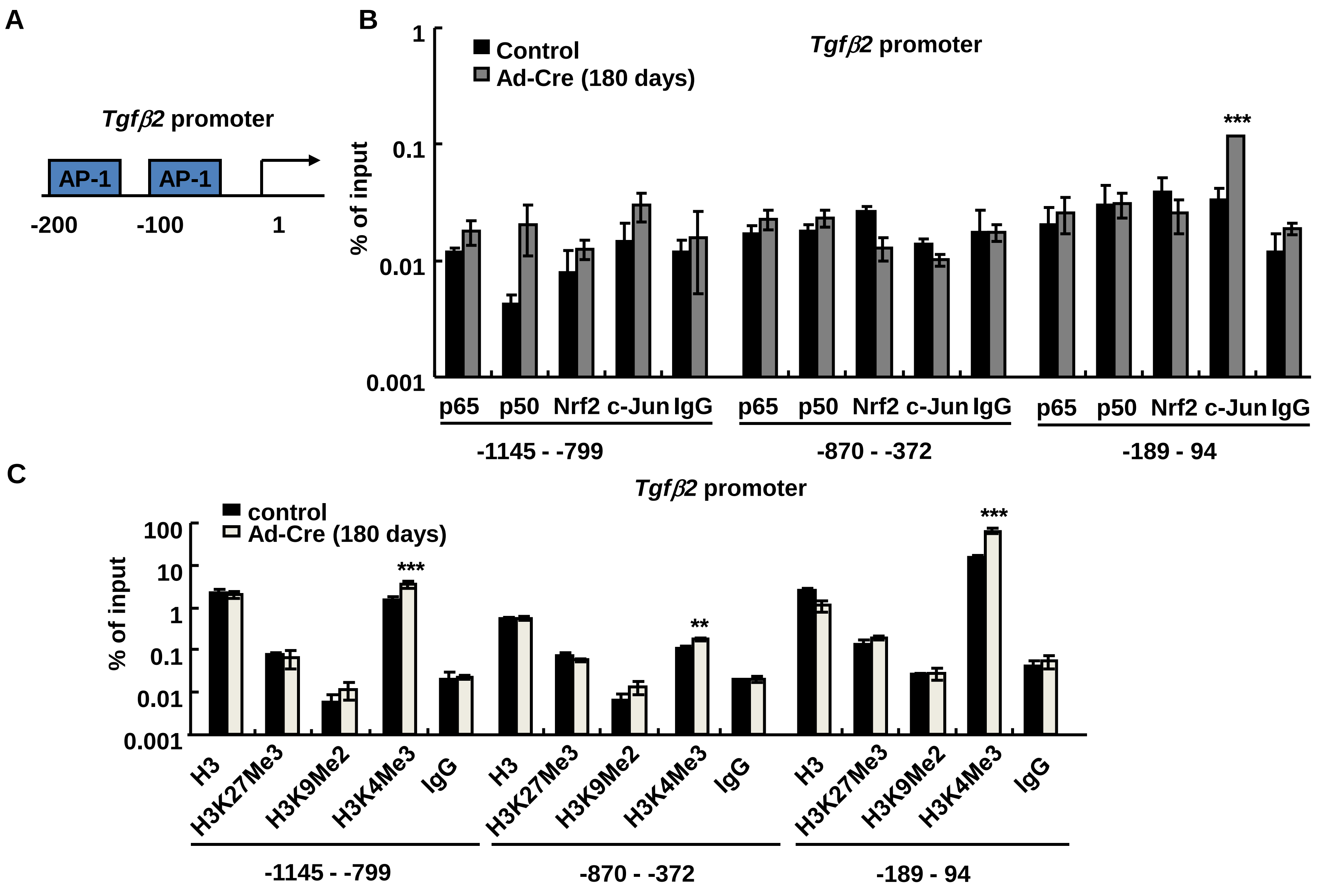

Chen, et. al. Supplemental Figure 6
